# Peroxisome Proliferator-Activated Receptor Gamma Modulator Promotes Neonatal Mouse Primordial Follicle Activation In Vitro

**DOI:** 10.3390/ijms21093120

**Published:** 2020-04-28

**Authors:** Sook Young Yoon, Ran Kim, Hyunmee Jang, Dong Hyuk Shin, Jin Il Lee, Dongwon Seol, Dong Ryul Lee, Eun Mi Chang, Woo Sik Lee

**Affiliations:** 1 Fertility Center of CHA Gangnam Medical Center, CHA University, Seoul 06125, Korea; 2 Department of Biomedical Science, CHA University, Seongnam-si 13488, Gyeonggi-do, Korea; 3 Department of Obstetrics and Gynecology, CHA University, Seoul 06125, Korea

**Keywords:** PPARγ, primordial follicle activation, ovarian reserve, PTEN/PI3K/AKT/FOXO3 pathway

## Abstract

Peroxisome proliferator-activated receptor gamma (PPARγ) is known as a regulator of cellular functions, including adipogenesis and immune cell activation. The objectives of this study were to investigate the expression of PPARγ and identify the mechanism of primordial follicle activation via PPARγ modulators in mouse ovaries. We first measured the gene expression of PPARγ and determined its relationship with phosphatase and tensin homolog (PTEN), protein kinase B (AKT1), and forkhead box O3a (FOXO3a) expression in neonatal mouse ovaries. We then incubated neonatal mouse ovaries with PPARγ modulators, including rosiglitazone (a synthetic agonist of PPARγ), GW9662 (a synthetic antagonist of PPARγ), and cyclic phosphatidic acid (cPA, a physiological inhibitor of PPARγ), followed by transplantation into adult ovariectomized mice. After the maturation of the transplanted ovaries, primordial follicle growth activation, follicle growth, and embryonic development were evaluated. Finally, the delivery of live pups after embryo transfer into recipient mice was assessed. While PPARγ was expressed in ovaries from mice of all ages, its levels were significantly increased in ovaries from 20-day-old mice. In GW9662-treated ovaries in vitro, PTEN levels were decreased, AKT was activated, and FOXO3a was excluded from the nuclei of primordial follicles. After 1 month, cPA-pretreated, transplanted ovaries produced the highest numbers of oocytes and polar bodies, exhibited the most advanced embryonic development, and had the greatest blastocyst formation rate compared to the rosiglitazone- and GW9662-pretreated groups. Additionally, the successful delivery of live pups after embryo transfer into the recipient mice transplanted with cPA-pretreated ovaries was confirmed. Our study demonstrates that PPARγ participates in primordial follicle activation and development, possibly mediated in part by the PI3K/AKT signaling pathway. Although more studies are required, adapting these findings for the activation of human primordial follicles may lead to treatments for infertility that originates from poor ovarian reserves.

## 1. Introduction

In mammals, the ovarian reserves formed during embryogenesis and the resulting nonrenewable, quiescent primordial follicles provide for an organism’s entire reproductive lifespan. While the majority of follicles remain dormant, a limited number of follicles are activated into growing follicles; when one dominant follicle reaches ovulation, the others undergo atresia in either the follicular stage or directly from the dormant stage [1]. The mechanism of maintaining the state of primordial follicles involves multiple local factors and intracellular signaling pathways; however, it remains ill-defined. Until recently, multiple activators including bone morphogenetic protein 4/7 (BMP4/7), growth differentiation factor 9 (GDF-9), Kit-ligand, fibroblast growth factor 2/7 (FGF2/7), insulin, gremlin 1/2 (GREM1/2), leukemia inhibitory factor (LIF) and suppressors including anti-Mullerian hormone (AMH), LIM homeobox 8 (LHX8), phosphatase and tensin homolog (PTEN), tuberous sclerosis complex 1(Tsc1)/mammalian target of rapamycin complex 1 (mTORC1), forkhead box O3a (FOXO3a), YAP (Hippo signaling), and forkhead box L2 (FOXL2) have been reported to be involved in primordial follicle development in various biochemical and genetic manipulation studies [2,3,4,5,6,7,8]. Among the pathways identified, recent work has focused on the PTEN/PI3K (phosphatidylinositol-3-kinase)/AKT (Protein kinase B)/FOXO3 signaling pathway. This pathway is known to regulate metabolism, cell growth, and cell survival. While considered a major cancer pathway, it is now clear that it also plays an important role in the regulation of dormancy and initial follicle activation in ovaries [9,10].

Previous studies using mutant mice have reported that the oocyte-specific deletion of PTEN caused global primordial follicle activation, similar to the phenotype induced by FOXO3a knock-out, and resulted in premature ovarian insufficiency [10,11]. These findings suggest that under-activation or negative regulation of the PTEN/PI3K/AKT/FOXO3 pathway may delay follicle activation and cause the excessive atresia of primordial follicles; thus, the inhibition of PTEN and the activation of PI3K may cause the activation of dormant follicles, since PTEN is known to negatively regulate the PI3K/AKT signaling pathway [12]. In this sense, the ability to recruit dormant follicles into the growing pool and support their complete development in vitro will facilitate the collection of oocytes available for assisted reproductive techniques and increase the effectiveness of ovarian tissue freezing to preserve female fertility. By contrast, the ability to sustain the dormancy of follicles would extend the female reproductive lifespan.

The transient use of PI3K stimulators has been found to temporarily accelerate primordial follicle activation and development in mouse and human ovaries [13]. Furthermore, previous studies have shown that the short term, the in vitro activation of dormant ovarian follicles via the stimulation of the PTEN/PI3K/AKT/FOXO3 signaling pathway with a PTEN inhibitor and a PI3K activator enhances primordial follicle activation [14], with improved oocyte maturation [12,15], suggesting that modulating the PI3K/AKT signaling pathway in patients with diminished ovarian reserves may have clinical utility [12]. However, a later study suggested that the inhibition of PTEN by a synthetic PTEN inhibitor may result in the activation of primordial follicles but could compromise the development of growing follicles [16]. Moreover, we should be cautious about the clinical applications of synthetic agents that lack any human teratogenicity data. Thus, it is important to investigate safe and effective activators of the PTEN/PI3K/AKT/FOXO3 signaling pathway.

Peroxisome proliferator-activated receptor gamma (PPARγ) was originally recognized as a regulator of cellular functions such as adipogenesis and immune cell activation [17]. However, recent articles have noted that PPARγ may also regulate cyclic changes in ovarian tissues [18,19]. Moreover, PPARγ was previously reported to positively control the expression of PTEN [20,21]. PPARγ is physiologically inhibited by cyclic phosphatidic acid (cPA), which is a naturally occurring phospholipid mediator and an analog of the growth factor-like phospholipid, lysophospholipid, a membrane phospholipid metabolite [22,23]. cPA is generated by the lysophospholipase D-catalyzed transphosphatidylation of lysophosphatidylcholine [24], which is mediated by cell surface G-protein-coupled receptors and PPARγ [22]. cPA acts as a second messenger to suppress PPARγ activation, induces a specific conformational change in PPARγ, and prevents agonist binding [24,25,26]. Considering that PPARγ agonists induce PTEN expression [27] and inhibit AKT phosphorylation in vitro [28], we hypothesized that treatment with PPARγ modulators, including cPA, may decrease both PTEN expression and the concomitant activation of primordial follicle growth.

The aim of this study was to investigate whether PPARγ modulators could induce the activation and growth of primordial follicles in 5-day-old female mice. We also wished to investigate whether the PTEN/PI3K/AKT/FOXO3 pathway could be regulated by a natural PPARγ antagonist. Finally, we wanted to elucidate the mechanism by which cPA suppresses PPARγ and affects primordial follicle activation.

## 2. Results

### 2.1. Gene Expression of PPARγ and PTEN in Mouse Ovaries

**Figure 1:**
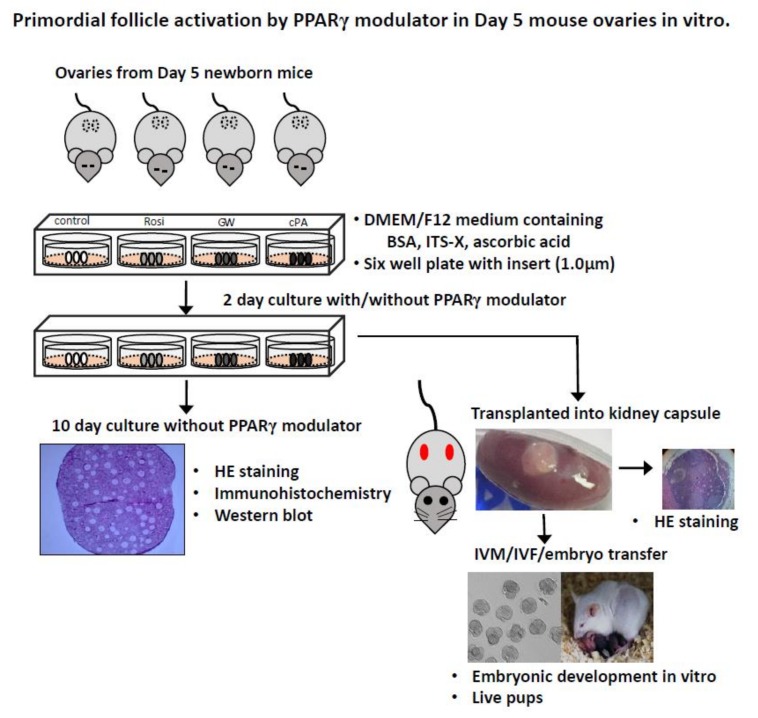
Schematic description of the experimental design. A cartoon shows in vitro co-culture with peroxisome proliferator-activated receptor gamma (PPARγ) modulators and ovarian transplantation with in vivo culture.

**Figure 2:**
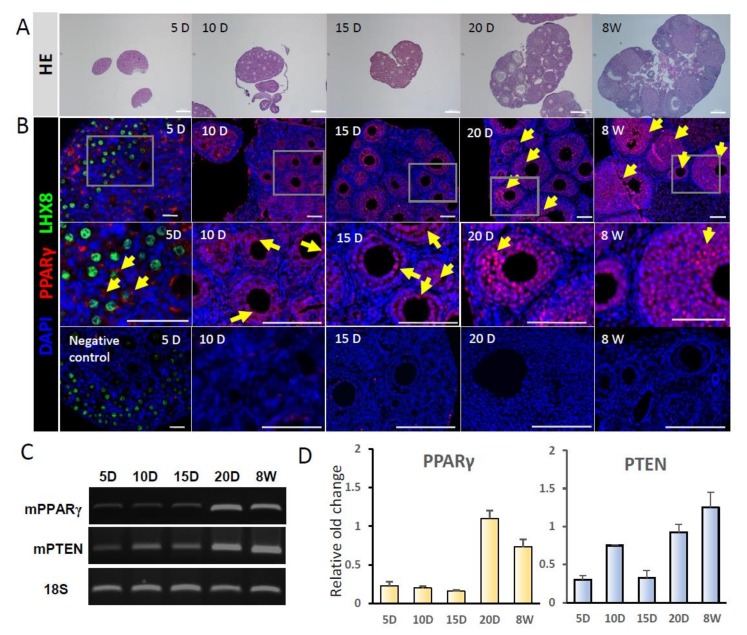
Identification of PPARγ in mouse ovaries. (**A**) Hematoxylin and eosin (HE) staining. Scale bar = 200 μm. (**B**) Immunostaining of PPARγ expression in 5-, 10-, 15-, and 20-day-old, and 8-week-old mouse ovaries. PPARγ staining is shown in red (yellow arrow); LIM homeobox 8 (LHX8) staining is shown in green. The negative control did not include the primary antibody against PPARγ. Scale bar = 100 μm. (**C**) RT-PCR analysis of gene expression. (**D**) Quantitation of results shown in (**C**).

A schematic description of the experiment is shown in Figure 1. Ovarian growth according to mouse age is shown in Figure 2a. To examine changes in the gene expression levels of PPARγ and PTEN with follicle development, RT-PCR and immunostaining were performed in ovaries from PD5, PD10, PD15, PD20, and 8W mice. PPARγ expression was localized in the cytoplasm of granulosa cells in PD5 mouse primordial follicles, in the nuclei of primary and secondary follicles in PD10 and PD15 mice, and in the antral follicles of PD20 and 8W mice; no expression was observed in stromal cells (Figure 2b). PPARγ and PTEN transcripts were detected in ovaries at all stages. Notably, the expression levels of PPARγ explosively increased in ovaries from PD20 and 8W mice (Figure 2c,d).

### 2.2. Effects on Primordial Follicle Activation and Development by PPARγ Modulators In Vitro

**Figure 3:**
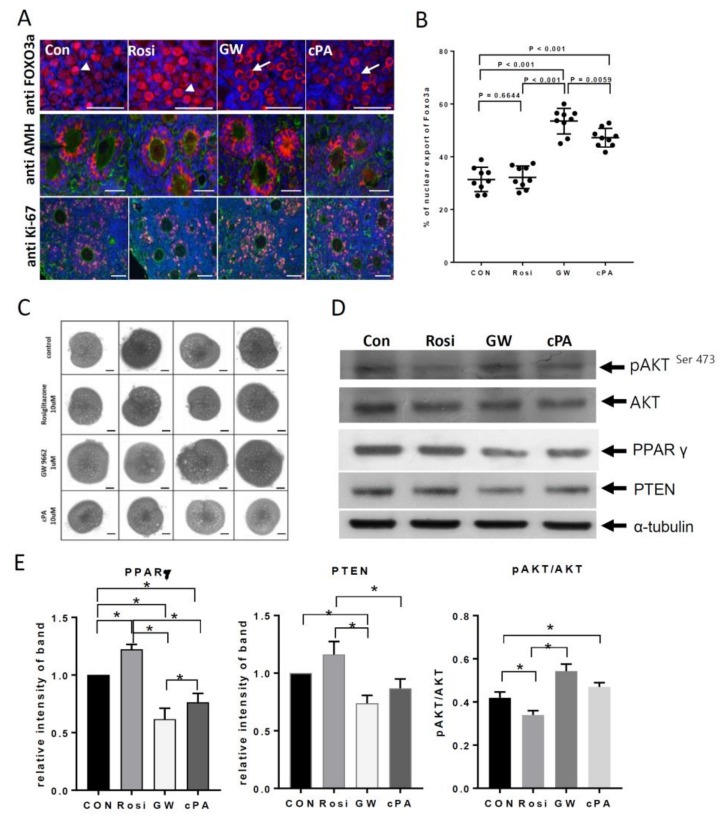
Primordial follicle activation with PPARγ modulation. (**A**) Nuclear exclusion of forkhead box O3a (FOXO3a) (red) in oocytes of primordial follicles 6 h after treatment with PPARγ modulators. Arrow heads point to FOXO3a located in the nuclei, whereas arrows point to FOXO3a located in the cytoplasm. Anti-Mullerian hormone (AMH) and Ki-67 staining are also shown in red, as indicated. Counterstaining was performed using anti-actin antibodies (green) and nucleus with DAPI (blue). Scale bar = 10 μm. (**B**) Percentages of primordial follicles with nuclear export of FOXO3a. (**C**) In vitro culture for 12 days of 5-day-old ovaries after 48 h treatment of PPARγ modulators. Scale bar = 100 μm. (**D**) Western blot analysis of ovaries after a 3-day treatment with PPARγ modulators. (**E**) Quantitation of results shown in (**D**). * indicates significant differences between groups, *p* < 0.05.

To determine the relationships between PPARγ, PTEN, AKT, and FOXO3a expression, neonatal mouse ovaries were treated with PPARγ modulators. Western blot analysis was performed to measure the relative expression of these proteins. In GW9662- and cPA-treated ovaries, the nuclear exclusion of FOXO3a was observed in oocytes in primordial follicles at 6 h (Figure 3a,b). To represent normal follicular growth in in vitro cultured ovaries, AMH expression in the cytoplasm and Ki-67 in the nucleus were detected in granulosa cells (Figure 3a). After 12 days of culture, the ovarian size in the GW9662- and cPA-treated groups was increased to that in the untreated and rosiglitazone-treated groups (Figure 3c). PPARγ and PTEN expression were increased and phosphorylated AKT was present at relatively low expression levels in rosiglitazone-treated ovaries. In GW9662- and cPA-treated ovaries, PTEN expression was decreased and phosphorylated AKT^Ser473^ was present at relatively high levels (Figure 3d,e). PPARγ and PTEN levels decreased after GW9662 treatment compared to with control or rosiglitazone treatment (Figure 3d,e, *p* < 0.05). Furthermore, AKT phosphorylation on Ser^473^ was significantly increased after GW9662 treatment (Figure 3d,e, *p* < 0.05).

**Figure 4:**
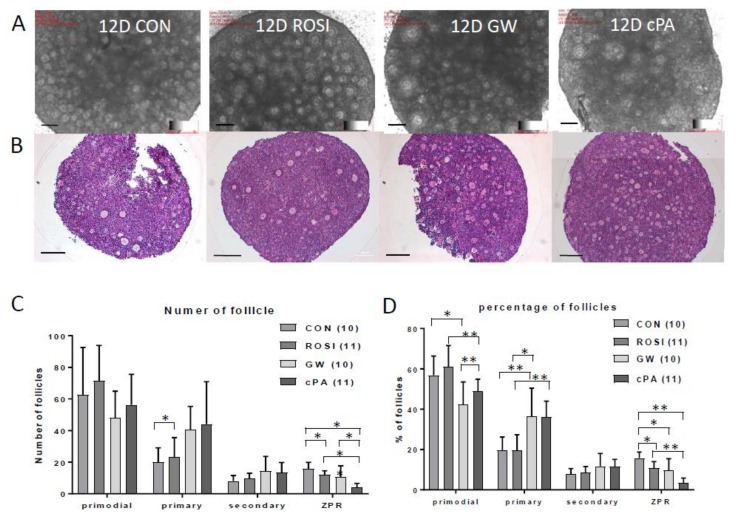
Ovarian histology showing follicle development at 12 days after treatment with PPARγ modulators. (**A**,**B**) Bright field and HE staining of ovaries from in vitro cultures of 5-day-old ovaries treated with PPARγ modulators for 12 days. Scale bar = 100 μm. Number of follicles (**C**) and percentage of follicles (**D**) in each stage are shown from HE staining. Numbers in parentheses of the legend indicate numbers of ovaries. ZPR, zona pellucida remnant. *, ** indicate significant differences between groups, *p* < 0.05, 0.001.

Histological sections of mouse ovaries showed an increase in the number of primary follicles in the GW9662- and cPA-treated groups (Figure 4). When differential counts of primordial, primary, secondary, and antral follicles were compared between groups, the mean number of follicles at each stage were not significantly different (Figure 4c). However, when differential ratios by percentages of each type of follicle were considered, GW9662 and cPA treatments were associated with a significantly higher number of primary follicles and a lower number of primordial follicles compared to the control group (Figure 4d, * *p* < 0.05, ** *p* < 0.001). Interestingly, cPA-treated ovaries showed a low ratio of zona pellucida remnants (ZPRs), which are markers of atresia (Figure 4d, * *p* < 0.05, ** *p* < 0.001).

### 2.3. Embryonic Development of Oocytes from Transplanted Ovaries

**Figure 5:**
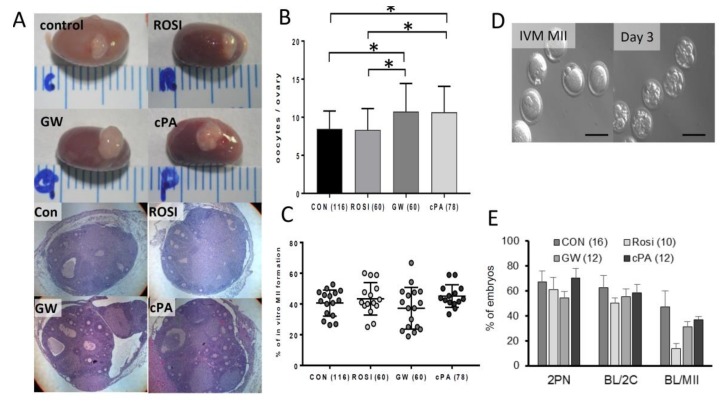
Ovarian histology showing follicle development after transplantation into kidney capsule. (**A**) Ovarian morphology 21 days after transplantation into the kidney capsule (upper) and HE staining (lower). (**B**) The number of oocytes per ovary from kidney transplantation and (**C**) the in vitro maturation (IVM) rate are shown. * indicates a significant difference between groups, *p* < 0.05. The numbers in parentheses represent the total numbers of transplanted ovaries. (**D**) Mature oocytes from IVM, 3-day-old embryos after intracytoplasmic sperm injection (ICSI), Scale bar = 100 μm, and (**E**) embryonic development after ICSI.

Twenty-one days after the transplantation of PPARγ modulator-treated ovaries, the numbers of oocytes, the ratio of polar body extrusion, fertilization, and the blastocyst formation rate were compared. Gross morphological findings revealed an expanded appearance of transplanted ovarian tissue after GW9662 and cPA pretreatment (Figure 5a). Additionally, significantly more oocytes were collected from the GW9662 group (average 10.7 ± 0.8) and the cPA group (10.6 ± 0.8) compared to from the control group (8.4 ± 0.5) and rosiglitazone group (8.3 ± 0.6, Figure 5b). However, there were no differences in the rate of oocyte maturation, fertilization, and the rate of embryonic development (Figure 5c–e).

### 2.4. Birth of Live Pups after Embryo Transfer into Recipient Mice

**Figure 6:**
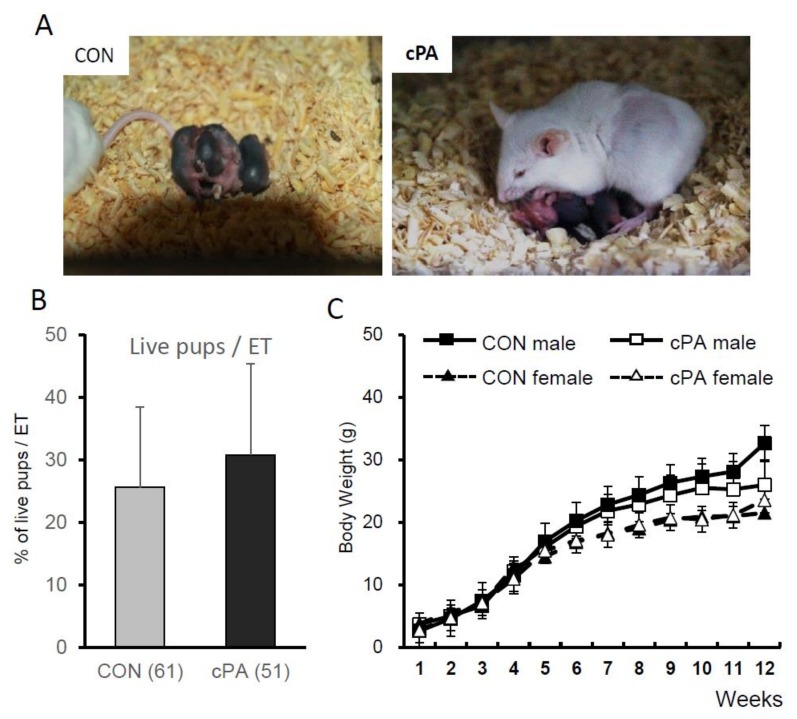
Birth of live pups after embryo transfer into recipient mice. (**A**) Live pups from control (CON) and cPA-treated ovaries. Sex ratios (**B**) and changes in body weight in live pups (**C**).

Two-cell embryos from IVF or intracytoplasmic sperm injection (ICSI) were transferred into the oviducts of pseudopregnant recipient mice, and the live birth rates were analyzed (Table 1, Figure 6a). Twenty offspring were delivered by three mice in the control group, 18 offspring were delivered by three mice in the cPA group, and changes in body weight were not significantly different between the groups (Figure 6b,c). Additionally, no newborn mice showed evidence of tumors, deformities, or developmental disorders.

Immature oocytes were collected from transplanted ovaries and matured in vitro, followed by in vitro fertilization. Embryos were transferred into 8-week-old pseudopregnant female mice.

## 3. Discussion

Attempts to activate primordial follicles may offer new opportunities for infertile couples, especially those at risk of diminished ovarian reserves, by increasing their oocyte recovery rate. Additionally, adolescent cancer patients may be able to preserve their fertility by effectively using cryopreserved ovarian tissue after cancer treatment. While PPARγ reportedly plays an important role in normal ovarian function, its role in primordial follicle activation has not been studied. In this study, we investigated the mechanism of primordial follicle activation using PPARγ modulators in mouse ovaries.

The expression of both PPARα and PPARδ is relatively stable throughout ovulation. On the other hand, PPARγ is detected early during folliculogenesis and is restricted primarily to granulosa cells in developing follicles, suggesting its role in the periodicity of follicle development [29,30]. The expression of PPARγ is downregulated in response to the surge in luteinizing hormone levels, which may induce negative feedback in follicle recruitment [31]. When PPARγ was specifically deleted using the Cre/loxP system in oocytes and granulosa cells, the estrous cycle was prolonged and fertility was reduced; however, the exact mechanism by which these effects occurred was not identified [32]. Additionally, the close relationship between PPARγ and polycystic ovary syndrome described in previous studies confirms the role of PPARγ in folliculogenesis [33,34,35,36]. Our results also indicate that PPARγ is highly functional in granulose cells; its ovarian expression was shown to be relatively low, peaking at PD20, which demonstrates a cyclic expression pattern that is suggestive of a role in controlling the reproductive cycle.

To explore the mechanism by which PPARγ affects folliculogenesis, specifically follicle recruitment, we used PPAR antagonists and agonists to evaluate a possible relationship between PPARγ and PTEN. PPARγ has been shown to positively regulate the expression of PTEN, which is a key regulator of primordial follicle activation [7,10,37,38,39]. From this point of view, we focused on evaluating the effects of PPARγ modulators, especially cPA, on primordial follicle activation. After cPA treatment, PTEN levels in PD5 ovaries decreased and AKT was activated, accompanied by the nuclear exclusion of FOXO3a. In turn, the activation of primordial follicles in neonatal mouse ovaries was stimulated. Increased AMH expression and an expanded morphology in PPARγ antagonist-treated ovaries shows a shift of primordial follicles to a growing pool.

Not only did we examine the effects of PPARγ modulators in vitro; we transplanted treated ovaries into recipient mice. After 1 month, cPA-pretreated, transplanted ovaries produced the highest numbers of oocytes and polar bodies, exhibited the most advanced embryonic development, and had the greatest blastocyst formation rate compared to the rosiglitazone- and GW9662-pretreated groups. The successful delivery of live pups with comparable survival after embryo transfer into recipient mice transplanted with cPA-pretreated ovaries was confirmed compared to control mice transplanted with untreated ovaries. This finding relieves safety concerns about cPA treatment. While comparable numbers of oocytes were collected from both cPA- and GW9662-treated ovaries, fewer live births were observed from mice transplanted with GW9662-treated ovaries. These findings suggest that cPA is a good candidate for primordial follicle activation.

Our current efforts show that PPARγ may participate in primordial follicle activation and development, possibly mediated in part by the PTEN/PI3K/AKT/FOXO3 signaling pathway in vitro. These results also suggest the efficacy of natural PPARγ antagonists in increasing the efficacy of oocyte recovery. However, epigenetic studies to evaluate offspring health were not done in this study, although normal litter size and fertility were observed. Additionally, since PPARγ dysregulation is closely associated with the development of metabolic diseases, such as impaired glucose tolerance and lipodystrophy [40], careful consideration should be taken before applying these findings clinically. Support from a larger study will inform the clinical use of PPARγ modulators—especially a physiological inhibitor of PPARγ, cPA—which have the potential to treat infertility originating from poor ovarian reserves.

## 4. Materials and Methods

### 4.1. Animals

Five-day-old female B6D2F1 mice, 8-week-old ICR female mice, and 12-week-old fertile male B6D2F1 mice were purchased from Samtako (Seoul, Korea), maintained in temperature- and humidity-controlled conditions under light (12 h) and dark (12 h) cycles, and fed a normal diet and water. All experiments described in this study were approved by the Institutional Animal Care and Use Committee (IACUC) of CHA University and performed in accordance with the guidelines presented by IACUC (Approval No. IACUC 140027 (2 March 2014)).

### 4.2. Gene Expression of PPARγ and PTEN in Mouse Ovaries

To evaluate the expression of PPARγ and PTEN in mouse ovaries, we performed real-time PCR and immunohistochemistry of 5-day-old (PD5), 10-day-old (PD10), 15-day-old (PD15), 20-day-old (PD20), and 8-week-old (8W) mouse ovaries. Total RNA from ovaries was isolated with TRIzol reagent (Invitrogen, Carlsbad, CA, USA) according to the manufacturer’s instructions. One microgram of good quality RNA was reverse transcribed to cDNA using a Prime Script RT reagent kit (TaKaRa Biotech, Kyoto, Japan). Quantitative real-time RT-PCR was carried out using an iCycler (Bio-Rad Laboratories Inc., Hercules, CA, USA) with the iQ SYBR Green Super mix (Bio-Rad) and iCycler iQ real-time detection system software. The reaction mixture consisted of 1 μL of cDNA, 1 µL of SYBR Green Super Mix, and 1 μL of primers (10 pmol/μL). PCR was performed using the following conditions: 40 cycles of denaturation at 95 °C for 40 s, annealing at 60 °C for 40 s, and extension at 72 °C for 40 s. Gene expression analysis of PPARγ and PTEN was performed in PD5, PD10, PD15, PD20, and 8W mouse ovaries using the following primers: PPARγ, 5′-gagtatgccaaaaatatccctggtttc-3′; and PTEN, 5′-agatatattcctccaattcaggaccca-3′. For immunohistochemistry, deparaffinized tissue sections were treated with antigen retrieval solution (Dako, Carpinteria, CA, USA) and blocked with a protein blocking solution (Dako) for 1 h at room temperature. The sections were incubated in primary antibodies against LHX8 (1:500, manufactured by Y.S. Choi) [41], PPARγ (1:100, Cat No. ab19481, Abcam, Cambridge, MA, USA), FOXO3a (1:100, Cat No. MA5-14932, Invitrogen), Ki-67 (1:100, Cat No. ab16667, Abcam), and AMH (1:100, Cat No. ab103233 Abcam) for 16 h at 4 °C, followed by incubation with diluted Alexa 488-conjugated anti-guinea pig antibody (for anti-LHX8, 1:500, Invitrogen) and Alexa 555-conjugated goat anti-rabbit antibody (Invitrogen) for 1 h at RT. The slides were washed in PBS/T three times for 5 min. Anti-actin antibodies (Chemicon International, Temecula, CA, USA) and DAPI (Invitrogen) were used for counter staining. The sections were observed and images were captured using an epifluorescence microscope (Axio Imager 2, Carl Zeiss, Oberkochen, Germany) with the ZEN imaging program. The negative control was the omission of primary antibody against PPARγ in PD5 and 8W mouse ovaries.

### 4.3. In Vitro Culture of Neonatal Mouse Ovaries with PPARγ Modulators

Paired ovaries were excised from PD5 B6D2F1 or ICR female mice and washed three times in M2 medium (Sigma-Aldrich, St. Louis, MO, USA) supplemented with 3 mg/mL of BSA (Sigma-Aldrich). Ovaries were cultured on inserts (1 µm, Thermo Fisher Scientific, Rockford, IL, USA) in a 6-well plate (Thermo Fisher Scientific) for 12 days in vitro. The culture medium, which consisted of DMEM/F12 (1:1, Gibco, Grand Island, NY, USA) containing 0.1% BSA (Sigma-Aldrich), 1% insulin-transferrin-selenium-X supplement (10 mg/mL of insulin, 5.5 mg/mL of transferrin, and 6.7 ng/mL of sodium selenite, Gibco), 0.05 mg/mL of L-ascorbic acid (Sigma-Aldrich), and antibiotics (50 U/mL of penicillin, 50 mg/mL of streptomycin, and 0.1% Albumax II; Gibco), was placed below the membrane insert to cover the ovaries. From a given donor, one ovary served as the control and the other was treated with PPARγ modulators (10 µM rosiglitazone, 1 µM GW9662, 10 µM cPA, Cayman Chemical, Ann Arbor, MI, USA) [24,25,42] for 48 h at 37 °C in a humidified atmosphere of 5% CO_2_ and further cultured for 10 days without PPARγ modulators. After 12 days, the ovaries were fixed and stained with hematoxylin and eosin (HE), and immunohistochemistry and Western blot experiments were performed.

For the Western blot analysis, 10 to 15 ovaries per group were washed in Dulbecco’s phosphate-buffered saline (Gibco) and lysed in PRO-PREP™ Protein Extraction Solution (Intron Biotechnology, Sungnam Si, Korea). After centrifugation at 13,000 rpm, the supernatant was diluted with 2× sample buffer (4% sodium dodecyl sulfate (SDS), 20% glycerol, 130 mM Tris-Cl, and 0.02% bromophenol blue) to 1 µg/µL and was frozen at 20 °C. The supernatants were subjected to SDS-polyacrylamide gel electrophoresis on 12% gels and transferred to a polyvinylidene difluoride membrane (Bio-Rad). The membrane was blocked with 5% nonfat dried milk or BSA (Bio-Rad) in 10 mM Tris (pH 7.4) and containing 0.1% Tween-20 for 1 h at room temperature. The membrane was then incubated with antibodies for PPARγ (1:1000, Cat No. ab19481, Abcam) and PTEN (1:1000, Cat No. 9559, Cell Signaling, Danvers, MA, USA). For the detection of phosphorylated AKT, the membrane was incubated with anti-phospho-AKT (1:1000, Cat No. 4051S, pSer^473^, Cell Signaling) at 4 °C, overnight with agitation. The membrane was then incubated with HRP-conjugated anti-mouse secondary antibody (Bio-Rad) for 1 h at room temperature. Peroxidase activity was visualized by enhanced chemiluminescence (ECL) using the Western Blotting Luminol Reagent (Santa Cruz Biotechnology Inc., Santa Cruz, CA, USA) and exposure to Amersham Hyperfilm ECL (GE Healthcare, Pittsburgh, PA, USA). Stripped membranes were reprobed with anti-AKT antibodies (1:1000, Cat No. 9272S, Cell Signaling) at 4 °C overnight. Visualized bands were quantified by densitometry using the NIH Image J software.

### 4.4. Ovarian Transplantation and In Vivo Culture

Recipient 8W female mice were anesthetized, and their kidneys were externalized through a dorso-horizontal incision. Paired ovaries (control and treatment groups) from the same donor were randomly inserted under the kidney capsule of the same recipient that was ovariectomized. One day after transplantation, hosts were treated daily with 2 IU of pregnant mare serum gonadotropin (PMSG, Sigma-Aldrich, [15]). Twenty-one days after transplantation, ovaries from recipient mice were collected and subjected to HE staining to evaluate ovarian growth.

### 4.5. In Vitro Maturation (IVM) and Embryonic Development of Oocytes from Transplanted Ovaries

Twenty-one days after transplantation, recipient mice were induced for superovulation by intraperitoneal injection with 5 IU of PMSG, and the grafted ovaries were collected 44 h later, in M2 medium. Ovaries were punctured with a 29-G needle. Germinal vesicle-stage oocytes were collected in Quinn’s advantage medium with HEPES (Sage In Vitro Fertilization, Inc., Trumbull, CT, USA) containing 10% serum protein substitute (Sage) and cultured for 16 h until reaching the second meiotic metaphase (MII), in TCM 199 medium containing 10% fetal bovine serum (Gibco), 0.075 IU/mL of recombinant follicle-stimulating hormone (Gonal F; Serono, Modugno Bari, Italy), and 0.5 IU/mL of hCG (Ovidrel; Serono). For in vitro fertilization, we performed in vitro fertilization (IVF) or intracytoplasmic sperm injection (ICSI) using sperm from the cauda epididymes of 10–12-week-old male mice, which had been incubated in Quinn’s Advantage Fertilization medium (Sage) for 30 min. Embryonic development after IVF/ICSI was evaluated by two pronuclear (2PN) and blastocyst formation on Day 5. Two-cell embryos, after IVM/IVF/ICSI, were transferred into the oviducts of pseudopregnant 8W female ICR mice. The births of live pups after embryo transfer into recipient mice were evaluated.

### 4.6. Statistical Analyses

All statistical analyses were performed using two-way ANOVA or Student’s *t* tests with GraphPad Prism 7 (GraphPad Software, Inc., La Jolla, CA, USA). *p* values lower than 0.05 or 0.001 were considered statistically significant.

## Figures and Tables

**Table 1:** Live birth results for cyclic phosphatidic acid (cPA)-treated ovaries used for embryo transfer into recipient mice.

	Control	cPA
Total number of embryos	61	57
Number of pseudo mother	9	7
Delivery mother	3	3
Live pup	20	18
